# Gender bias in the clinical reasoning steps of medical students: a critical examination

**DOI:** 10.1186/s12909-026-09184-w

**Published:** 2026-04-17

**Authors:** Joana Le Boudec, Gina Potarca, Sylvie Félix, Joëlle Schwarz, Margot Guth, Carole Clair

**Affiliations:** 1 Health and Gender Unit, Unisanté, University Center for Primary Care and Public Health & University of Lausanne, Lausanne, Switzerland; 2 Department of Sociology, Social Policy and Criminology, School of Law and Social Justice, University of Liverpool, Liverpool, UK; 3 Faculty of Biology and Medicine, Clinical Skills Unit, Medical School, University of Lausanne, Lausanne, Switzerland

**Keywords:** Gender, Bias, Stereotypes, Medical student, Medical education, Objective Structured Clinical Examination, Clinical reasoning

## Abstract

**Background:**

Despite growing awareness of the importance of integrating gender knowledge into medical education, gender stereotypes persist and may influence patient assessment and management. This study investigates gender inequalities in clinical reasoning among medical students to identify areas for improvement in medical education.

**Methods:**

The study was conducted at the University of Lausanne in Spring 2021, using the Objective Structured Clinical Examination (OSCE) to assess fifth-year medical students. Students were evenly assigned to interactions with either a male or female standardised patient (SP) presenting with unintentional weight loss. Evaluation covered history taking, physical examination, and clinical management. A total of 105 students (57.1% female, 42.9% male) were assessed.

**Results:**

Results indicate potential gender bias at various stages of clinical reasoning, with patterns depending on the gender of both the SP and the student. During history-taking, female students were less likely to ask female SPs about alcohol consumption than male SPs (56.3% vs. 78.6%, *p* = 0.07). Regarding occupational history, a compelling trend was also observed among male students, who asked female SPs less often (30.4% vs. 59.1%, *p* = 0.05), whereas female students showed more consistent rates. Additional compelling trends emerged during physical examinations: male students performed cardiac auscultation less often on female SPs (56.5% vs. 86.4%, *p* = 0.02). Although diagnostic hypotheses and differential diagnoses were similar, female SPs were more often prescribed laboratory tests (63.6% vs. 26.0%, *p* < 0.001).

**Conclusions:**

Gender bias permeates multiple stages of clinical reasoning among medical students, leading to under-recognition of key health risk factors, differences in examination thoroughness, and increased prescription of laboratory tests in female patients. Addressing gender bias through sustained integration of gender into core medical education is essential for diagnostic accuracy and high-quality patient care. Specifically, systematic inquiry into occupational and alcohol histories in female patients, improved cardiovascular auscultation, and enhanced communication with male patients are needed.

**Supplementary Information:**

The online version contains supplementary material available at 10.1186/s12909-026-09184-w.

## Background

In recent years, promoting health equity for all, including gender minorities and women, has become increasingly prominent in global health policy agenda, notwithstanding persistent challenges [1]. This study uses the term sex to refer to the biological characteristics based on sexual phenotype and chromosomes, and gender to refer to a multi-faceted concept encompassing socially constructed roles, behaviours, and expressions [2, 3]. Gender is a critical social determinant of health, intersecting with factors such as race, socioeconomic status (SES), and age to shape health inequities. Following the SAGER (Sex and Gender Equity in Research) Guidelines [4] and prior studies on gender stereotypes [5, 6], we use “gender” here to address gender bias in clinical reasoning related to role and behavioural.

Medicine has historically been shaped by androcentric norms that position the male body as the “standard”, contributing to gender inequities in research and care [7, 8]. Gender inequalities in healthcare manifest through bias at various steps of the medical system, from education to access to care, including gender biases among health practitioners and patients [8, 9]. Gender blindness - the failure to recognise gender-specific needs and experiences - and gender stereotypes are major drivers of gender bias in healthcare [10]. These negative implicit (i.e., unconscious) biases are particularly concerning in clinical settings, where they affect patient care. A better understanding of biases in clinical reasoning could help clinicians reduce clinical errors and improve patient safety [11].

The biomedical literature on implicit bias in healthcare has explored biases related to race, class, gender, and age. A 2017 review found that healthcare professionals exhibit implicit biases at similar levels to the general population [12] contributing to health disparities. However, most implicit bias research uses indirect measures, such as the Implicit Association Test (IAT) [13], clinical vignettes, or a combination of both [14]. Some social and human sciences studies use qualitative methods to directly observe how biases influence clinical decisions. For instance, Swiss research revealed implicit biases in clinical reasoning among medical students, such as tendency to enquire about female patients’ family situation and about male patients’ occupational situation [15], or unprotected sex [16].

While qualitative findings provide valuable insights into the nature and manifestation of implicit bias, more quantitative studies are needed to measure its occurrence at different stages of clinical reasoning. Some, including our previous observation, showed students were less likely to diagnose anxiety disorders in men despite identical symptoms presentation [5]. More research is needed to fully capture their scope and extent. To our knowledge, this study is one of the few to directly and quantitatively investigate implicit biases in clinical reasoning.

### Research question/aim

This study specifically aims to understand if and how patient and student gender influence the attitudes and actions of medical students through the different steps of a clinical consultation. Additionally, the study aims to collect context-specific quantitative evidence on the occurrence and manifestations of gender biases in patient management, providing insights for medical education activities.

## Methods

### Study design and population

This quantitative study was integrated into a summative assessment of clinical competencies using an Objective Structured Clinical Examination (OSCE). The study population consisted of fifth-year medical students (*N* = 210, 123 female) at the University of Lausanne, Switzerland, who underwent the OSCE as part of their mandatory exam. In the spring of 2021, half (105 out of 210) participated in the study, randomly assigned to the circuit containing the scenario relevant to our research question. Due to logistical constraints, two OSCE circuits with different stations were run on separate days.

### Objective Structured Clinical Examination (OSCE)

For the OSCE, students individually managed a standardised patient (SP) - an actor playing a patient according to a structured script - while being evaluated by an examiner. The same script was applied to all students to ensure equal assessment of performance. Students were assessed on history-taking, physical examination, diagnostic and therapeutic management using station-specific checklists. Communication skills were evaluated with the Analytic Global OSCE Rating [17], consisting of four items on responding to patient needs, interview structure, and verbal and non-verbal expression.

The OSCE comprised five stations, each lasting 13 min, divided into two parts: the first involved a patient encounter, and the second a post-encounter station with an expert in which management and diagnoses were discussed.

### Outcome(s) and pre-specified hypotheses

The primary outcome was the students’ evaluation on items of history-taking, physical examination, proposed investigations, diagnosis, communication and subsequent management through the different steps of a clinical consultation, according to the SP gender.

We hypothesized that patient gender, and its interaction with student gender, would influence specific process-level components of clinical reasoning, including history-taking (e.g., greater inquiry into family in female SPs and occupational history in male SPs), assessment of risk factors (e.g., smoking status more frequently explored in male SPs), physical examination, communication patterns, and management decisions (e.g., chest radiography more frequently requested for male SPs).

### Case vignette and Standardised Patients (SPs)

This study was integrated into one of the OSCE stations, focusing on a patient with unintended weight loss. Half of the students encountered a female SP, and the other half a male SP. Apart from gender, all other patient characteristics were similar. Before entering the examination room, students received case vignettes (Table 1). The SPs were trained to respond based on their scripts, which included additional details about symptoms, habits, personal and family history, and social situation.

**Table 1:** Description of the case vignette for students

You are doing an internship in a family doctor's office as an assistant doctor. A 57-year-old female/male patient presents to the family practice for unintentional weight loss. The patient is known to the office, and the doctor asks you to start seeing the patient on your own and to pass on the results of the consultation later. During the examination, the patient's vital signs were recorded as follows: temperature: 36.5°C, blood pressure: 130/65 mmHg, and pulse: 85 beats/per minute.	

According to the SP script, students encountered a 57-year-old patient in a private family medicine practice, presenting with unintentional weight loss (10 kg/6 months), fatigue, and a usual cough that had recently worsened. The patient, an active smoker, had a history of depression, generalised anxiety, and stage II chronic obstructive pulmonary disease (COPD), as well as a family history of stroke (mother) and head and neck cancer (father). The only medication was salbutamol on demand (approximately 2–3 x/week). The SP was married for 30 years, had 3 children and 2 grandchildren, worked in her/his own clothing store and had a medium SES. S/he felt stressed by the weight loss. During the physical examination, the patient appeared thin but fit, with normal vital signs. The only finding was on thoracic examination: percussion showed large pulmonary fields without dullness, and distant but symmetrical vesicular breath sounds on auscultation. Biological findings were non-specific, and chest X-ray showed a small opacity with blurry external boundaries, in the shape of a grass fire, located at the left apex.

In the first part of the station, students questioned and examined the SP. In the second, they were expected to summarise their findings in terms of medical history and examination, propose differential diagnosis, request ancillary tests, and suggest a final diagnosis once the test results were available. The final expected diagnosis was pulmonary neoplasia.

### Assessment of the case vignette

Station-specific checklists (Appendices 1 and 2) were created for assessing students during their exam. The case vignette and checklist were internally reviewed within the Clinical Skills Unit (SF) to ensure content validity, clarity of items, and alignment with curricular objectives. Each checklist contained specific items, followed by overall assessments for each step of the evaluation. In accordance with the standardised OSCE setting, trained examiners directly observed the student-SP encounter and completed the structured checklists, adhering strictly to predefined items, supported by senior OSCE leads to ensure consistent application of the scoring criteria. SP training was standardised and conducted by the same trainer; the quality of role-play was checked during the exam. Students, SPs, and examiners were blinded to the study’s purpose.

### Data collection

Data were obtained from the completed station-specific checklists. To ensure participant confidentiality, only de-identified data were used for analysis.

### Statistical methods

We compared the single checklist item scores of the students who evaluated female versus male SPs, using chi-squared tests for each item. Scores were also compared separately by SP gender for male and female students. Statistical analysis was performed using STATA 14, with a significance threshold of *p* < 0.05; however, all p-values were reported transparently. Findings in small, stratified subgroups (e.g., male students with female SPs) that reached this threshold were nevertheless highlighted in the Results section as “compelling trends” rather than definitive evidence, given the limited sample size and the risk of Type I and Type II errors [18]. Trends that did not reach statistical significance (*p* ≥ 0.05) were also highlighted, in line with recommendations to avoid strictly dichotomous interpretations of statistical significance [19].

### Training on gender bias

Before this summative assessment, all medical students at the University of Lausanne received a two-hour compulsory introductory course on gender medicine in their first year, and a few registered for a follow-up optional seminar on gender medicine (18 students in 2017, 16 women and 2 men). They also received a one-hour compulsory course in their fourth year introducing the topic of gender and pain, including gender bias in pain management. Since 2019, first-year master’s students in medicine participate in the Gender Reflexivity Project [15], a clinically integrated module enhancing reflexivity on how gender influences clinical management. Weekly small group discussions (4–5 students) with a clinical supervisor and a gender medicine expert encouraged reflection on cases encountered during the outpatient clinic. This study included students who had all been exposed to this module.

## Results

### Population

The study population is summarised in Table S1. A total of 105 students were included (45 male (42.9%), 60 female (57.1%)). They were exposed to the same clinical vignette, with half encountering a male SP (50 encounters, 47.6%) and half a female SP (55 encounters, 52.4%). Eight examiners assessed the students.

Results with *p* ≥ 0.05 are reported as trends, as they do not reach conventional statistical significance. The process-level differences are presented below by successive steps of a clinical consultation, as they may inform targeted educational and assessment strategies in medical training.

### First part of the station

#### History-taking

**Fig. 1:**
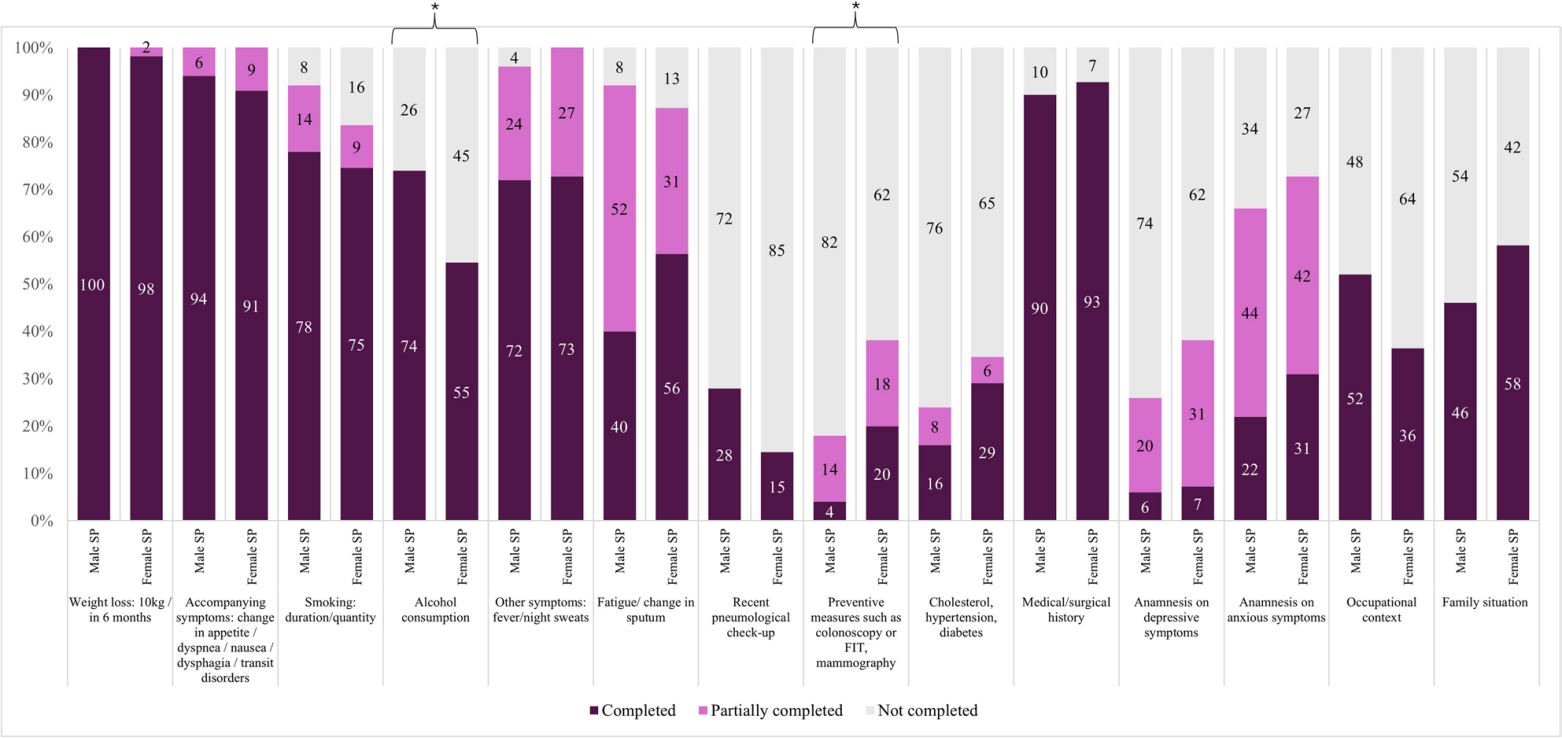
History-taking items asked, by standardised patient (SP) gender Male SP: Male standardised patient, Female SP: Female standardised patient, FIT: fecal immunochemical test * *p* < 0.05. See Appendix 1 for more details on content and evaluation criteria

Differences in information collected during history-taking depended on both SP and student gender (Fig. 1, Table S2). Overall, students were less likely to ask women about alcohol consumption than men (54.6% vs. 74.0% male SPs, *p* = 0.04). This was mainly observed among female students, who showed a trend towards asking female SPs less often (56.3% vs. 78.6% male SPs, *p* = 0.07). Among male students, a trend towards asking female SPs less about occupational context was observed (30.4% vs. 59.1% male SPs, *p* = 0.05), while female students’ rates were similar across SP genders (40.6% vs. 46.4% male SPs, *p* = 0.65).

Students were also more likely to ask female SPs about preventive measures, such as colonoscopy/FIT or mammography (20.0% vs. 4.0% male SPs, *p* = 0.03), and tended to inquire more about fatigue or sputum changes (56.4% vs. 40.0% male SPs, *p* = 0.09). Conversely, women tended to be asked less about prior pulmonary controls, such as radiographs and pulmonary functions (14.6% vs. 28.0% male SPs, *p* = 0.09).

Questions about weight loss, supplementary symptoms, smoking habits, vaccines, comorbidities, medical and surgical history were asked equally often. History-taking of depressive, anxious symptoms and family situation were also similar.

Examiners’ global evaluations of history-taking were similar for female or male SPs (*p* = 0.90). However, male students tended to perform poorer history-taking with female SPs (13.0% rated as well performed vs. 40.9% male SPs, *p* = 0.07) (Figure S1: Part A).

#### Physical examination

**Fig. 2:**
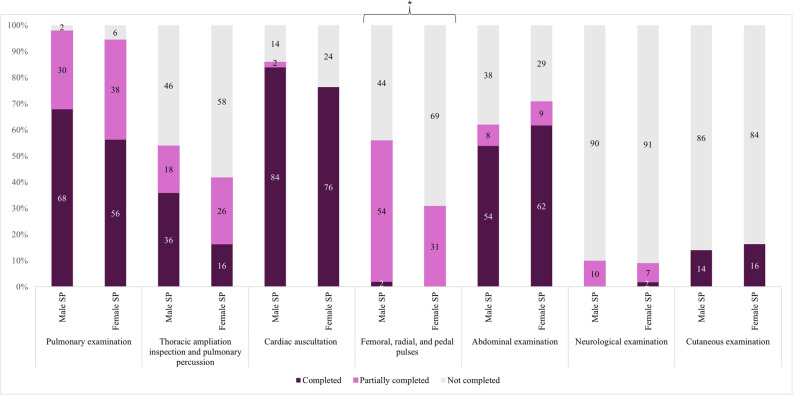
Physical examination items performed, by standardised patient (SP) gender Male SP: Male standardised patient, Female SP: Female standardised patient. * *p* < 0.05. See Appendix 1 for more details on content and evaluation criteria

 During auscultation, a compelling trend was observed: female SPs received fewer cardiac auscultations from male students (56.5% vs. 86.4% male SPs, *p* = 0.02) (Fig. 2, Table S3). When the student was female, cardiac auscultations were similar for female and male SPs (90.6% vs. 82.1% male SPs, *p* = 0.34). Femoral, radial, and pedal pulses were taken less often in female SPs (*p* = 0.03), a difference mainly observed among male students (*p* = 0.05). Additionally, women tended to receive chest expansion inspection and pulmonary percussion less often than men (16.4% vs. 36.0% male SPs, *p* = 0.07). Female SPs received similar pulmonary, abdominal, neurological, and cutaneous examinations to male SPs.

Global evaluation (Figure S1: Part B) suggested female SPs were less well examined (*p* = 0.08), with a compelling trend driven by male students (auscultation rated well: 4.4% vs. 45.5% male SPs, *p* < 0.001). In contrast, female students showed similar performance across SP genders (37.5% vs. 32.1% male SPs, *p* = 0.53).

#### Communication

Global communication was rated higher with female SPs (*p* = 0.02), with a compelling trend observed among female students (*p* = 0.01) (Figure S2). Female students also showed a compelling trend toward better interview structure with female SPs (*p* = 0.02) and tended to respond better to the feelings and needs of women (*p* = 0.11). In contrast, male students tended to have better verbal communication with male SPs (*p* = 0.08).

### Second part of the station

#### Clinical management (presentation of history-taking and status, and clinical decision making)

**Fig. 3:**
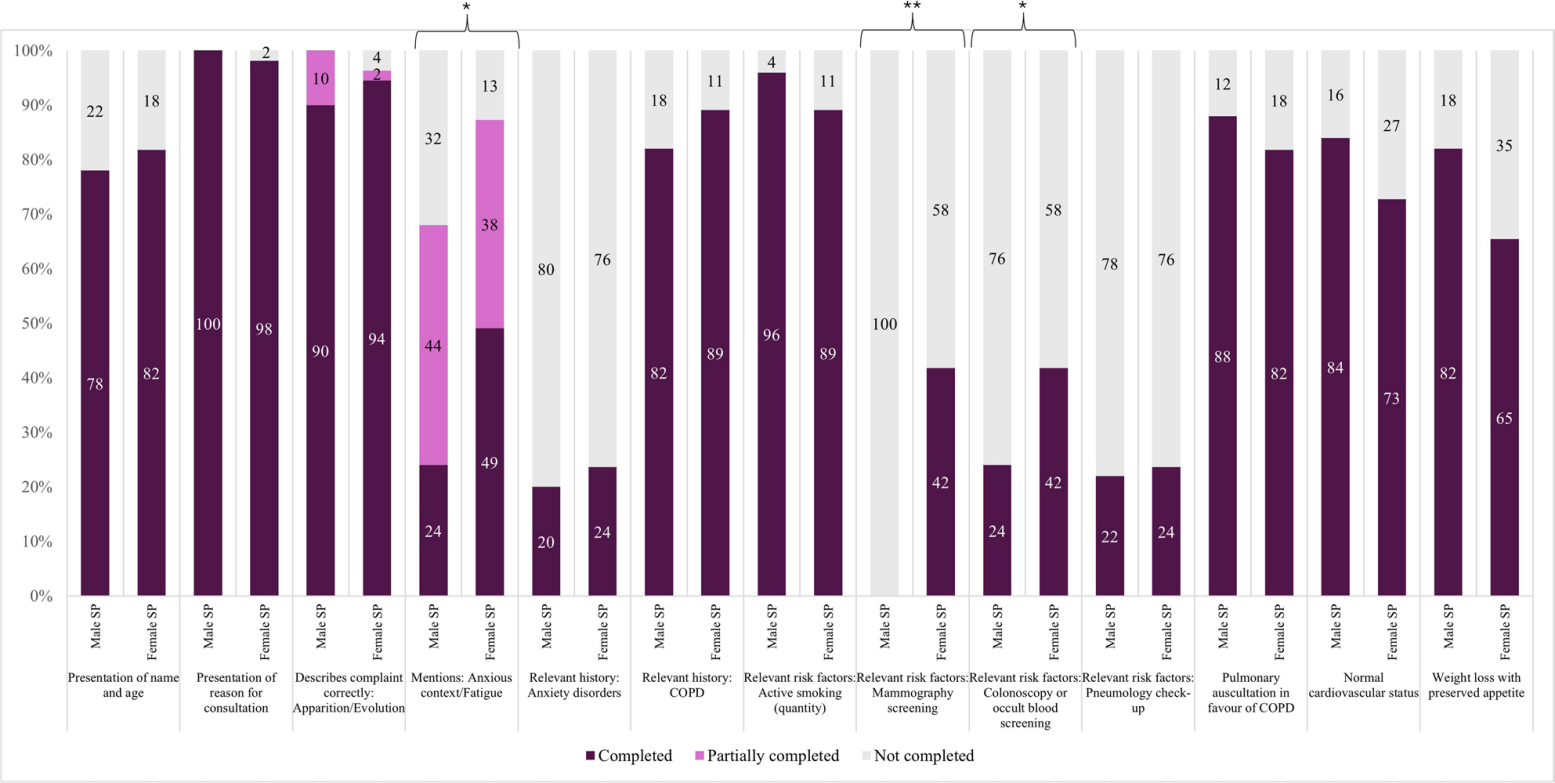
Clinical management (presentation of history-taking and status), by standardised patient (SP) gender Male SP: Male standardised patient, Female SP: Female standardised patient. * *p* < 0.05; ** *p* < 0.001. See Appendix 2 for more details on content and evaluation criteria

Students tended to report weight loss characteristics less often for female SPs (65.5% vs. 82.0% male SPs, *p* = 0.06), a trend primarily observed among female students (68.7% vs. 89.3% male SPs, *p* = 0.05) (Fig. 3, Table S4). Anxious context and fatigue were reported more often for female SPs (49.1% vs. 24.0% male SPs, *p* = 0.01), as were preventive measures such as mammography, as expected (*p* < 0.001), and colonoscopy (*p* = 0.05), with a compelling trend among female students (40.6% vs. 14.3% male SPs, *p* = 0.02). Female students additionally showed a compelling trend toward underreporting active smoking in female SPs (81.2% vs. 100.0% male SPs, *p* = 0.02). Presentation of name and age, reason for consultation, onset and evolution of main symptom, past pulmonary controls and history of anxiety disorder and COPD were similar. The physical examination was presented similarly, except for cardiovascular status, which tended to be presented more often for male SPs, particularly by male students (56.5% vs. 81.8% male SPs, *p* = 0.07). Global evaluation of the patient presentation was similar (*p* = 0.87) (Figure S3).

 Diagnostic hypothesis and differential diagnoses were comparable (pulmonary neoplasia: 76.4% vs. 78.0% male SPs, *p* = 0.62), with similar arguments used (weight loss, smoking habit, absence of recent thoracic imagery) (Table S5). However, laboratory tests were prescribed more frequently for female SPs (***p*** < 0.001), with a compelling trend among female students (68.8% vs. 14.3% male SPs, *p* < 0.001). In contrast, imaging requests were similar (*p* = 0.71), and the final diagnosis of pulmonary neoplasia following the imagery report was made in 100% of female and 98% of male SPs, reflecting the OSCE design, which revealed the radiological diagnosis when requested. Overall, clinical reasoning and global management were similar across SP genders (*p* = 0.16).

Overall, post-encounter communication (Figure S4) showed better structure and linearity for female SPs (***p*** = 0.003), with a compelling trend among male students (*p* = 0.006). Verbal communication tended to be better with female SPs (*p* = 0.06), with a similar trend among female students (*p* = 0.05). The global evaluation on communication similarly favoured female SPs (*p* < 0.001).

## Discussion

This study highlights the occurrence of gender bias at various steps of clinical reasoning and management among medical students. While several findings are consistent with previous quantitative [5, 20, 21], and qualitative studies [15, 16, 22, 23], this study also allows us to explore where and how gender bias unfolds throughout successive phases of a clinical consultation. These findings provide evidence to address bias in medical education.

### Biased clinical reasoning steps

During history-taking, the first step in clinical consultation, some results showed gender bias. Students were less likely to ask women about alcohol consumption. This discrepancy may stem from two key factors. First, the gendered historical and social representation that alcohol consumption is primarily a male behaviour could lead students to ask male patients more often. Second, the higher prevalence of alcohol dependence among men in Switzerland may explain differences in investigations, despite narrowing gender difference [24, 25]. Not investigating women’s alcohol use contributes to the under-diagnosis of an addiction that is even more stigmatised in this gender, closing a window of opportunity to address the problem [26]. Identifying this bias can help raise awareness of implicit biases in gender and alcohol use, and other risk behaviours. From an educational perspective, this finding highlights the importance of reflexive approaches that encourage students to critically examine how social representations may influence what they consider clinically relevant to ask.

Students, particularly male students, tended to enquire less often about women’s previous pulmonary investigations and occupational history, consistent with qualitative studies [15, 16], and may reflect prevailing Swiss social representations of the gender division of labour (i.e., men in formal, full-time jobs, women in the reproductive sphere) [27]. Women were asked slightly more often about preventive measures. However, the results on preventive measures cannot be interpreted clearly because mammography and colonoscopy/FIT were asked and evaluated together. In Switzerland, mammography is performed regularly as part of organised screening, for women from the age of 50 onwards, and the SP was 57, it was expected that female SP be asked about this measure [28]. Combining gender-specific (mammography) and non-gender-specific (colonoscopy/FIT) components may have diluted observed differences, potentially underestimating the full extent of gender bias in preventive care.

During the physical examination, findings suggest that male students were less likely to perform cardiac auscultation when examining female SPs, whereas no comparable pattern was observed among female students. Consistent with prior research describing cardiac auscultation as a gender-sensitive examination [29], our observations support the notion that examination performance may be influenced by student-patient gender interactions. Several explanations have previously been proposed, including anatomical considerations over the mitral area and discomfort related to the sexualisation of female breasts, which may intimidate male students [29]. This omission is clinically relevant, as early cardiac assessment is essential for timely diagnosis and intervention - an area in which women face well-documented delays [30]. Although these omissions did not affect the final diagnosis in our controlled OSCE, they could have greater consequences in real-world practice where diagnostic tests are not automatically available. This suggests the importance of supervised, gender-sensitive physical examination training, combined with guided reflexivity on discomfort or implicit avoidance behaviours, and their influence on clinical practice. Furthermore, femoral, radial, and pedal pulses were assessed less often in women than in men, contrary to previous observations [5]. Because pulse examinations in the different regions were assessed together, it was difficult to discern assessment differences in more sensitive areas, such as the inguinal region, which may have masked the true extent of gender bias.

Communication was rated higher with female patients, especially by female students, consistent with previous findings [31–33]. This aligns with gendered cultural norms impoverishing communication in men due to adherence to hegemonic masculine norms (e.g., restrictive emotionality, preoccupation with power and competition, maintaining dominance by hiding weaknesses) [34]. This bias needs to be addressed through communication skills training and a reflection on gendered assessment of communication. Awareness and understanding of the impact of the clinician’s communication style on the exchange of information with patients can enhance treatment adherence, treatment outcomes, and overall patient satisfaction [33].

In the second step, where students summarised findings, female patients again received greater attention for preventive measures, signs of anxiety and/or fatigue. However, female students also appeared to underreport active smoking and weight loss in female patients. A French study similarly noted that tobacco use was more frequently discussed by general practitioners with male patients [35], potentially reflecting persistent stereotypes that associate smoking predominantly with male behaviour [36]. Although gender differences in smoking prevalence have decreased - in Switzerland, the smoking rate among women is even now slightly higher in the 15–24 age group [37] - women are still considered to be less likely to smoke. These findings suggest that gender bias may also operate at the stage of clinical synthesis, influencing which information is emphasized or omitted in diagnostic narratives. Such patterns could be addressed in case-based learning to help students recognize and mitigate biases. This issue is compounded by the fact that many clinical guidelines are still primarily based on cohorts of white men, whose risk factors may not accurately reflect those of women or individuals from diverse ethnic and socio-economic backgrounds [38]. While prevalence rates can guide differential diagnoses, they should not preclude the investigation of less commonly considered risk factors, such as alcohol or tobacco use among women.

Contrary to previous findings [39], laboratory tests were requested more often for female patients, possibly reflecting broader questioning of women’s diagnoses due to greater attention to psychosomatic symptoms, such as anxiety and fatigue. This aligns with higher identification of anxiety in women [5], putting men at risk of under-recognition [40]. Additionally, imagery was asked for similarly. A Swiss study on acute low back pain management found women referred for imaging less often than men [6], suggesting men’s complaints are taken more seriously.

Differences in clinical reasoning and actions did not affect diagnosis, which was similar for male and female SPs, reflecting the OSCE design where radiology results were automatically provided if requested. This design constraint prevented the observed process biases from translating into an observable diagnostic error. Thus, the study focuses on process biases in history-taking, examination, and communication, rather than diagnostic errors.

Finally, participants had completed a practical course on implicit gender bias a year before the OSCE. While the course raised awareness on gender bias using a reflective approach [15], gender-related biases persisted in clinical reasoning and examination. As highlighted by Gonzales et al., singular teaching sessions are unlikely to produce sustainable change in clinical practice. Rather, implicit bias recognition and management “must be re-framed as an epistemology of practice that is essential to the professional identity of medical learners to be effective” [41]. However, this course represents an important first step in raising awareness, uncommon in other medical curricula, that needs further consolidation to achieve lasting impact. Taken together, our findings indicate that gender bias may emerge at multiple stages of the consultation process, highlighting the need for sustained, longitudinal reflective practice in clinical training to support critical reflection and equitable assessment of patient information.

### Strength and limitations

A strength of this study is the standardised conditions of social determinants (age, SES, ethnicity), which allowed isolation of gender effect on otherwise identical vignettes. Additionally, students, SPs, and examiners were all blinded to the study’s purpose, crucial for studying implicit bias. This study also provides quantitative evidence of gender-related biases across successive stages of the clinical consultation, complementing and extending previous qualitative findings [15].

Nevertheless, this study is not without limitations. The small sample size may reduce the statistical power, and some observations arose from particularly small strata and should therefore be viewed as exploratory rather than conclusive findings. We reported and discussed observed trends when they were consistent with existing evidence or suggested potential practical relevance. Such trends should be interpreted cautiously but may still offer meaningful insights for future research and medical education [19]. Examiner gender was not considered due to variation between morning and afternoon sessions and small subgroup sizes combining examiner, student, and SP gender. Similar to previous studies [5, 42, 43], this research involved final-year medical students, which prevented us from assessing differences in attitudes toward gender-related medical issues between first- and final-year students, or the influence of clinical experience on gender sensitivity. Some students attended an optional gender medicine seminar, which may have provided additional exposure to gender-related topics. However, this concerned less than 5% of the study sample and is therefore unlikely to have meaningfully influenced our findings. Lastly, our study is limited to one University centre in Lausanne, where interventions on gender implicit bias are implemented. Further studies in different contexts are needed to confirm and strengthen our findings on one hand, and to assess if and how the integration of the gender bias module may influence student’s skills. Cultural and institutional characteristics specific to the medical training context in Lausanne may limit generalizability.

## Conclusion

These findings provide essential evidence for medical education and for activities raising awareness of when and how implicit bias may occur in a clinical encounter [11]. By recognising and mitigating these biases, healthcare providers will sustain diagnostic accuracy, ensure equitable care, and improve patient outcomes. Addressing gender bias in clinical reasoning and practice is essential. Our study identifies specific gender biases requiring attention and intervention, including the need to systematically explore occupational history and drinking habits in female patients. Despite recent improvements, further efforts are also needed in teaching cardiovascular auscultation in female patients at both pre- and post-graduate physicians. Additionally, it appears that physicians need to reflect further on their communication with men.

## Supplementary Information

Supplementary Material 1.

## Data Availability

The datasets used and/or analysed during the current study are available from the corresponding author on reasonable request. Restrictions apply to the case vignettes described thoroughly in the text, which are only available after permission of the Faculty of Biology and Medicine of the University of Lausanne.

## Appendix 1

### First part of the station: vignette case - content and evaluation criteria.

For the first part of the station, students were required to take a medical history and perform a targeted clinical examination on either a female or a mal SP. They were evaluated based on the following criteria:

1. History-taking (14 items)
  - Content:
    - ◦ Characteristics of weight loss (10 kg/6 months)
    - ◦ Accompanying symptoms (change in appetite, dyspnoea, nausea, dysphagia, transit disorders
    - ◦ Smoking (duration and quantity)
    - ◦ Alcohol consumption
    - ◦ Other symptoms (fever, night sweats)
    - ◦ Fatigue and change in sputum
    - ◦ Recent pulmonary check-up
    - ◦ Preventive measures such as colonoscopy or FIT, mammography
    - ◦ Cholesterol, hypertension, diabetes
    - ◦ Medical/surgical history
    - ◦ History-taking on depressive symptoms
    - ◦ History-taking on anxious symptoms
    - ◦ Occupational context
    - ◦ Family situation)
  - Evaluation:
    - ◦ Each item was considered: “completed” (2 points), “partially completed” (1 point), or “not completed” (0 points)
    - ◦ Alcohol consumption, recent pulmonary check-up, medical/surgical history, occupational context, and family situation were evaluated as either: “completed” (2 points) or “not completed” (0 points)
    - ◦ An overall assessment of the history-taking (focused, logical flow) was also conducted and rated as: “good” (2 points), “sufficient” (1 point), or “insufficient” (0 points)
2. Physical examination (7 items)
  - Content:
    - ◦ Pulmonary examination
    - ◦ Chest expansion inspection and pulmonary percussion
    - ◦ Cardiac auscultation
    - ◦ Femoral, radial, and pedal pulses
    - ◦ Abdominal examination
    - ◦ Neurological examination
    - ◦ Cutaneous examination
  - Evaluation:
    - ◦ Each item was considered: “completed” (2 points), “partially completed” (1 point), or “not completed” (0 points)
    - ◦ Pulmonary and cutaneous examination were evaluated as either: “completed” (2 points) or “not completed” (0 points)
    - ◦ An overall assessment of the physical examination was also conducted and rated as: “good” (2 points), “sufficient” (1 point), or “insufficient” (0 points)

3. Communication: Doctor-patient relationship (4 items).
  - Content:
    - ◦ Responding to patients’ feelings and needs
    - ◦ Interview structure
    - ◦ Verbal expression
    - ◦ Non-verbal expression
  - Evaluation:
    - ◦ Each item was assessed on a scale from 1 (not at all) to 5 (totally)
    - ◦ An overall assessment of the communication was also conducted and rated: from 1 (incompetent) to 5 (exceptionally competent)

## Appendix 2

### Second part of the station: vignette case - content and evaluation criteria

For the second part, students were required to *present* their clinical management, including the presentation of history-taking and status, as well as clinical decision-making, in presence of an expert, but without SP. They were evaluated based on the following criteria:

1. Clinical management (13 items)
  - Content:
    - ◦ Presentation of name and age
    - ◦ Presentation of reason for consultation
    - ◦ Correctly describes the complaint: onset/evolution
    - ◦ Mentions: anxiety context/fatigue
    - ◦ Relevant history: anxiety disorders
    - ◦ Relevant history: COPD
    - ◦ Relevant risk factors:
      - ▪ Active smoking (quantity)
      - ▪ Mammography screening
      - ▪ Colonoscopy or occult blood screening
      - ▪ Pulmonary check-up
    - ◦ Pulmonary auscultation in favour of COPD
    - ◦ Normal cardiovascular status
    - ◦ Weight loss with preserved appetite
  - Evaluation:
    - ◦ Each item was considered: “completed” if mentioned (2 points), or “not completed” (0 points),
    - ◦ The correct description of the complaint (onset/evolution) and mentions of anxiety context/fatigue were evaluated as: “completed” for mention of both (2 points), “partially completed” for mention of one of the two (1 point), or “not completed” for none (0 points).
    - ◦ An overall assessment of the patient's presentation - focusing on its relevance and coherence - was also conducted and rated as: “good” (2 points), “sufficient” (1 point), or “insufficient” (0 points).
2. Diagnostic elements and clinical reasoning
  After the presentation of the case, which summarised the relevant information from the medical history and physical examination, students were asked to answer five questions:
  i. “What is your main diagnostic hypothesis?”:
    - Pulmonary neoplasia (2 points)
    - Other neoplasia (1 point)
    - Not relevant (0 point)
  ii. “Are there any alternative diagnoses (1-2) that you have thought of?”:
    - Neoplasia or COPD (2 points)
    - Depression or decompensated anxiety disorder (1 point)
    - None or not relevant (0 point)
  iii. “Can you cite the main arguments that led you to propose these diagnostic hypotheses?”
    - *▪ Main arguments expected*: Significance of weight loss/Smoking habits/Absence of recent chest imaging.
      - At least 2 arguments (2 points)
      - 1 argument (1 point)
      - None or not relevant (0 point)
  iv. “What happens next in the diagnostic process?”
    - *▪ Expected laboratory tests*: Blood count, TSH, creatinine, liver tests.
      - At least 3 arguments (2 points)
      - 1 or 2 arguments (1 point)
      - None or not relevant (0 point)
    - *▪ Imaging expected*: Chest X-ray, abdominal scan, chest scan
      - At least 2 arguments (2 points)
      - 1 argument (1 point)
      - None or not relevant (0 point)
      After discussing the diagnostic approach, students were given the laboratory and chest X-ray results.
  v. “What is your final diagnosis?”
    - Pulmonary neoplasia (2 points)
    - Excludes endocrinology or hemato-oncology (1 point)
    - Not relevant (0 point).
    - Evaluation: An overall assessment of clinical reasoning was also conducted and rated as “good” (2 points), “sufficient” (1 point), or “insufficient” (0 points)
3. Evaluation of post-encounter communication (2 items)
  - Content:
    - ◦ Structure and linearity of presentation: Summarise the case in a structured, linear, logical, and easily understandable way
    - ◦ Verbal language: Transcribe the information provided by the patient using appropriate and correct medical terminology.
  - Evaluation:
    - ◦ Both items were rated from 1 (not at all) to 5 (totally)
    - ◦ An overall assessment of the post-encounter communication was also conducted and rated: from 1 (incompetent) to 5 (exceptionally competent)

## Abbreviations

OSCE: Objective structured clinical examination
SPs: Standardised patients
