# Supplementary material for: Gender bias in the clinical reasoning steps of medical students: a critical examination
Source: BMC Med Educ. 2026 Apr 17;26:867. doi: 10.1186/s12909-026-09184-w (PMC13220609; doi:10.1186/s12909-026-09184-w)
Supplement: Supplementary file 1 — Supplementary Material 1. [file 12909_2026_9184_MOESM1_ESM.docx]

**Table S1**. Characteristics of study populations

| **Characteristics** | Total | Women | Men |
| --- | --- | --- | --- |
|  | N | N (%) | N (%) |
| Students | 105 | 60 (57.1) | 45 (42.9) |
| Standardised patients | 8 | 4 (50.0) | 4 (50.0) |
| **First part of the station** |  |  |  |
| Morning examiners | 4 | 3 (75.0) | 1 (25.0) |
| Afternoon examiners | 4 | 2 (50.0) | 2 (50.0) |
| **Second part of the station** |  |  |  |
| Morning examiners | 4 | 2 (50.0) | 2 (50.0) |
| Afternoon examiners | 4 | 1 (25.0) | 3 (75.0) |

**Table S2.** Anamnesis items asked, by standardised patient (SP) gender and by student gender.

| **Evaluation item: Anamnesis** |  | Male student | | | Female student | | |
| --- | --- | --- | --- | --- | --- | --- | --- |
|  |  | Male SP (%) | Female SP (%) | p-value^*^ | Male SP (%) | Female SP (%) | p-value^*^ |
| Weight loss: 10kg / in 6 months | Completed | 100.0 | 100.0 | 1.0 | 100.0 | 96.9 | 0.35 |
|  | Partially completed | 0.0 | 0.0 |  | 0.0 | 3.1 |  |
|  | Not completed | 0.0 | 0.0 |  | 0.0 | 0.0 |  |
|  |  |  |  |  |  |  |  |
| Accompanying symptoms: change in appetite/dyspnea/nausea/dysphagia/transit disorders | Completed | 95.5 | 91.3 | 0.58 | 92.9 | 90.6 | 0.76 |
|  | Partially completed | 4.5 | 8.7 |  | 7.1 | 9.4 |  |
|  | Not completed | 0.0 | 0.0 |  | 0.0 | 0.0 |  |
|  |  |  |  |  |  |  |  |
| Smoking: duration/quantity | Completed | 72.7 | 82.6 | 0.64 | 82.1 | 68.7 | 0.28 |
|  | Partially completed | 18.2 | 8.7 |  | 10.7 | 9.4 |  |
|  | Not completed | 9.1 | 8.7 |  | 7.2 | 21.9 |  |
|  |  |  |  |  |  |  |  |
| Alcohol consumption | Completed | 68.2 | 52.2 | 0.27 | 78.6 | 56.2 | 0.07 |
|  | Partially completed | 0.0 | 0.0 |  | 0.0 | 0.0 |  |
|  | Not completed | 31.8 | 47.8 |  | 21.4 | 43.8 |  |
|  |  |  |  |  |  |  |  |
| Other symptoms: fever/night sweats | Completed | 68.2 | 65.2 | 0.53 | 75.0 | 78.1 | 0.56 |
|  | Partially completed | 27.3 | 34.8 |  | 21.4 | 21.9 |  |
|  | Not completed | 4.5 | 0.0 |  | 3.6 | 0.0 |  |
|  |  |  |  |  |  |  |  |
| Fatigue/ change in sputum | Completed | 40.9 | 52.2 | 0.38 | 39.3 | 59.4 | 0.21 |
|  | Partially completed | 50.0 | 30.4 |  | 53.6 | 31.2 |  |
|  | Not completed | 9.1 | 17.4 |  | 7.1 | 9.4 |  |
|  |  |  |  |  |  |  |  |
| Recent pneumological check-up | Completed | 31.8 | 13.0 | 0.13 | 25.0 | 15.6 | 0.37 |
|  | Partially completed | 0.0 | 0.0 |  | 0.0 | 0.0 |  |
|  | Not completed | 68.2 | 87.0 |  | 75.0 | 84.4 |  |
|  |  |  |  |  |  |  |  |
| Preventive measures such as colonoscopy or FIT, mammography | Completed | 4.6 | 13.0 | 0.60 | 3.6 | 25.0 | **0.02** |
|  | Partially completed | 27.3 | 26.1 |  | 3.6 | 12.5 |  |
|  | Not completed | 68.2 | 60.9 |  | 92.8 | 62.5 |  |
|  |  |  |  |  |  |  |  |
| Cholesterol, hypertension, diabetes | Completed | 18.2 | 17.4 | 0.86 | 14.3 | 37.5 | 0.13 |
|  | Partially completed | 13.6 | 8.7 |  | 3.6 | 3.1 |  |
|  | Not completed | 68.2 | 73.9 |  | 82.1 | 59.4 |  |
|  |  |  |  |  |  |  |  |
| Medical/surgical history | Completed | 90.9 | 87.0 | 0.67 | 89.3 | 96.9 | 0.24 |
|  | Partially completed | 0.0 | 0.0 |  | 0.0 | 0.0 |  |
|  | Not completed | 9.1 | 13.0 |  | 10.7 | 3.1 |  |
|  |  |  |  |  |  |  |  |
| Anamnesis on depressive symptoms | Completed | 4.5 | 8.7 | 0.34 | 7.1 | 6.3 | 0.84 |
|  | Partially completed | 18.2 | 34.8 |  | 21.4 | 28.1 |  |
|  | Not completed | 77.3 | 56.5 |  | 71.4 | 65.6 |  |
|  |  |  |  |  |  |  |  |
| Anamnesis on anxious symptoms | Completed | 18.2 | 30.4 | 0.50 | 25.0 | 31.3 | 0.85 |
|  | Partially completed | 36.4 | 39.1 |  | 50.0 | 43.7 |  |
|  | Not completed | 45.4 | 30.5 |  | 25.0 | 25.0 |  |
|  |  |  |  |  |  |  |  |
| Occupational context | Completed | 59.1 | 30.4 | **0.05** | 46.4 | 40.6 | 0.65 |
|  | Partially completed | 0.0 | 0.0 |  | 0.0 | 0.0 |  |
|  | Not completed | 40.9 | 69.6 |  | 53.6 | 59.4 |  |
|  |  |  |  |  |  |  |  |
| Family situation | Completed | 50.0 | 56.5 | 0.66 | 42.9 | 59.4 | 0.20 |
|  | Partially completed | 0.0 | 0.0 |  | 0.0 | 0.0 |  |
|  | Not completed | 50.0 | 43.5 |  | 57.1 | 40.6 |  |

*P-values of chi-squared tests for each evaluation item. Bold indicates p <0.05.
Male SP: Male standardised patient, Female SP: Female standardised patient.
See **Appendix 1** for more details on content and evaluation criteria.

**Part B**

**Part A**

**

**Figure S1**. Global evaluation of students' anamnesis (Part A) and physical examination (Part B), rated good, sufficient, or insufficient.
** p < 0.001
See **Appendix 1** for more details on content and evaluation criteria.

**Table S3.** Physical examination items performed, by standardised patient (SP) gender and by student gender.

| **Evaluation item: Physical examination** | | Male student | | | Female student | | |
| --- | --- | --- | --- | --- | --- | --- | --- |
|  |  | Male SP (%) | Female SP (%) | p-value^*^ | Male SP (%) | Female SP (%) | p-value^*^ |
| Pulmonary examination | Completed | 72.7 | 69.6 | 0.36 | 64.3 | 46.9 | 0.37 |
|  | Partially completed | 27.3 | 21.7 |  | 32.1 | 50.0 |  |
|  | Not completed | 0.0 | 8.7 |  | 3.6 | 3.1 |  |
|  |  |  |  |  |  |  |  |
| Thoracic ampliation inspection and pulmonary percussion | Completed | 27.3 | 4.4 | 0.11 | 42.9 | 25.0 | 0.33 |
|  | Partially completed | 22.7 | 30.4 |  | 14.3 | 21.9 |  |
|  | Not completed | 50.0 | 65.2 |  | 42.8 | 53.1 |  |
|  |  |  |  |  |  |  |  |
| Cardiac auscultation | Completed | 86.4 | 56.5 | **0.02** | 82.1 | 90.6 | 0.34 |
|  | Partially completed | 4.5 | 0.0 |  | 0.0 | 0.0 |  |
|  | Not completed | 9.1 | 43.5 |  | 17.9 | 9.4 |  |
|  |  |  |  |  |  |  |  |
| Femoral, radial, and pedal pulses | Completed | 0.0 | 0.0 | **0.05** | 3.6 | 0.0 | 0.15 |
|  | Partially completed | 54.6 | 26.1 |  | 53.5 | 34.4 |  |
|  | Not completed | 45.4 | 73.9 |  | 42.9 | 65.6 |  |
|  |  |  |  |  |  |  |  |
| Abdominal examination | Completed | 54.6 | 56.5 | 0.99 | 53.6 | 65.6 | 0.47 |
|  | Partially completed | 13.6 | 13.0 |  | 3.5 | 6.3 |  |
|  | Not completed | 31.8 | 30.5 |  | 42.9 | 28.1 |  |
|  |  |  |  |  |  |  |  |
| Neurological examination | Completed | 0.0 | 0.0 | 0.52 | 0.0 | 3.1 | 0.64 |
|  | Partially completed | 9.1 | 4.4 |  | 10.7 | 9.4 |  |
|  | Not completed | 90.9 | 95.6 |  | 89.3 | 87.5 |  |
|  |  |  |  |  |  |  |  |
| Cutaneous examination | Completed | 9.1 | 13.0 | 0.67 | 17.9 | 18.8 | 0.93 |
|  | Partially completed | 0.0 | 0.0 |  | 0.0 | 0.0 |  |
|  | Not completed | 90.9 | 87.0 |  | 82.1 | 81.2 |  |

*P-values of chi-squared tests for each evaluation item. Bold indicates p <0.05.
Male SP: Male standardised patient, Female SP: Female standardised patient.
See **Appendix 1** for more details on content and evaluation criteria.

**Figure S2**. Global evaluation of students' communication (scale from 1 (not at all) to 5 (totally))
* p < 0.05
See **Appendix 1** for more details on content and evaluation criteria.

*

*

*

**Table S4.** Clinical management (presentation of anamnesis and status), by standardised patient (SP) gender and by student gender.

| **Evaluation criteria: Clinical management** | | Male student | | | Female student | | |
| --- | --- | --- | --- | --- | --- | --- | --- |
|  |  | Male  SP (%) | Female SP (%) | p-value^*^ | Male  SP (%) | Female SP (%) | p-value^*^ |
| Presentation of name  and age | Completed | 68.2 | 87.0 | 0.13 | 85.7 | 78.1 | 0.45 |
|  | Partially completed | 0.0 | 0.0 |  | 0.0 | 0.0 |  |
|  | Not completed | 31.8 | 13.0 |  | 14.3 | 21.9 |  |
|  |  |  |  |  |  |  |  |
| Presentation of reason for consultation | Completed | 100.0 | 100.0 | 1.0 | 100.0 | 96.9 | 0.35 |
|  | Partially completed | 0.0 | 0.0 |  | 0.0 | 0.0 |  |
|  | Not completed | 0.0 | 0.0 |  | 0.0 | 3.1 |  |
|  |  |  |  |  |  |  |  |
| Describes complaint correctly: Onset/Evolution | Completed | 90.9 | 91.3 | 0.51 | 89.3 | 96.9 | 0.11 |
|  | Partially completed | 9.1 | 4.4 |  | 10.7 | 0.0 |  |
|  | Not completed | 0.0 | 4.4 |  | 0.0 | 3.1 |  |
|  |  |  |  |  |  |  |  |
| Mentions:  Anxiety context/Fatigue | Completed | 22.7 | 52.2 | 0.11 | 25.0 | 46.9 | 0.07 |
|  | Partially completed | 50.0 | 34.8 |  | 39.3 | 40.6 |  |
|  | Not completed | 27.3 | 13.0 |  | 35.7 | 12.5 |  |
|  |  |  |  |  |  |  |  |
| Relevant history: Anxiety disorders | Completed | 22.7 | 26.1 | 0.79 | 17.9 | 21.9 | 0.70 |
|  | Partially completed | 0.0 | 0.0 |  | 0.0 | 0.0 |  |
|  | Not completed | 73.3 | 73.9 |  | 82.1 | 78.1 |  |
|  |  |  |  |  |  |  |  |
| Relevant history: COPD | Completed | 86.4 | 91.3 | 0.60 | 78.6 | 87.5 | 0.36 |
|  | Partially completed | 0.0 | 0.0 |  | 0.0 | 0.0 |  |
|  | Not completed | 13.6 | 8.7 |  | 21.4 | 12.5 |  |
|  |  |  |  |  |  |  |  |
| Relevant risk factors:  Active smoking (quantity) | Completed | 90.9 | 100.0 | 0.14 | 100.0 | 81.2 | **0.02** |
|  | Partially completed | 0.0 | 0.0 |  | 0.0 | 0.0 |  |
|  | Not completed | 9.1 | 0.0 |  | 0.0 | 18.8 |  |
|  |  |  |  |  |  |  |  |
| Relevant risk factors: Mammography screening | Completed | 0.0 | 34.8 | 0.002 | 0.0 | 46.9 | **<0.001** |
|  | Partially completed | 0.0 | 0.0 |  | 0.0 | 0.0 |  |
|  | Not completed | 100.0 | 65.2 |  | 100.0 | 53.1 |  |
|  |  |  |  |  |  |  |  |
| Relevant risk factors: Colonoscopy or occult blood screening | Completed | 36.4 | 43.5 | 0.63 | 14.3 | 40.6 | **0.02** |
|  | Partially completed | 0.0 | 0.0 |  | 0.0 | 0.0 |  |
|  | Not completed | 63.6 | 56.5 |  | 85.7 | 59.4 |  |
|  |  |  |  |  |  |  |  |
| Relevant risk factors: Pneumology check-up | Completed | 27.3 | 13.0 | 0.23 | 17.9 | 31.3 | 0.23 |
|  | Partially completed | 0.0 | 0.0 |  | 0.0 | 0.0 |  |
|  | Not completed | 72.7 | 87.0 |  | 82.1 | 68.7 |  |
|  |  |  |  |  |  |  |  |
| Pulmonary auscultation in favour of COPD | Completed | 86.4 | 78.3 | 0.48 | 89.3 | 84.4 | 0.58 |
|  | Partially completed | 0.0 | 0.0 |  | 0.0 | 0.0 |  |
|  | Not completed | 13.6 | 21.7 |  | 10.7 | 15.6 |  |
|  |  |  |  |  |  |  |  |
| Normal cardiovascular status | Completed | 81.8 | 56.5 | 0.07 | 85.7 | 84.4 | 0.89 |
|  | Partially completed | 0.0 | 0.0 |  | 0.0 | 0.0 |  |
|  | Not completed | 18.2 | 43.5 |  | 14.3 | 15.6 |  |
|  |  |  |  |  |  |  |  |
| Weight loss with  preserved appetite | Completed | 72.7 | 60.9 | 0.40 | 89.3 | 68.7 | **0.05** |
|  | Partially completed | 0.0 | 0.0 |  | 0.0 | 0.0 |  |
|  | Not completed | 27.3 | 39.1 |  | 10.7 | 31.3 |  |

*P-values of chi-squared tests for each evaluation item. Bold indicates p <0.05.
Male SP: Male standardised patient, Female SP: Female standardised patient.
See **Appendix 2** for more details on content and evaluation criteria.

**Figure S3**. Global evaluation of the patient's presentation, rated good, sufficient, or insufficient.
See **Appendix 2** for more details on content and evaluation criteria.

**Table S5.** Diagnostic elements and clinical reasoning, by standardised patient (SP) gender and by student gender.

| **Diagnostic elements and clinical reasoning** | | Total student (N=105) | | | Male student (N=45) | | | Female student (N=60) | | |
| --- | --- | --- | --- | --- | --- | --- | --- | --- | --- | --- |
|  |  | Male  SP (%) | Female SP (%) | p-value^*^ | Male  SP (%) | Female SP (%) | p-value^*^ | Male  SP (%) | Female SP (%) | p-value^*^ |
| Main diagnostic hypothesis | Pulmonary neoplasia | 78.0 | 76.4 | 0.62 | 63.6 | 78.3 | 0.12 | 89.3 | 75.0 | 0.27 |
|  | Other neoplasia | 6.0 | 10.9 |  | 4.6 | 13.0 |  | 7.1 | 9.4 |  |
|  | Not relevant | 16.0 | 12.7 |  | 31.8 | 8.7 |  | 3.6 | 15.6 |  |
|  |  |  |  |  |  |  |  |  |  |  |
| Differential diagnosis | Neoplasia or COPD | 90.0 | 90.9 | 0.78 | 95.4 | 95.6 | 0.97 | 85.7 | 87.5 | 0.75 |
|  | Depression or decompensated anxiety disorder | 8.0 | 5.5 |  | 4.6 | 4.4 |  | 10.7 | 6.3 |  |
|  | None or not relevant | 2.0 | 3.6 |  | 0.0 | 0.0 |  | 3.6 | 6.2 |  |
|  |  |  |  |  |  |  |  |  |  |  |
| Main arguments: Significance of weight loss/Smoking habits/Absence of recent chest imaging | At least 2 | 80.0 | 74.6 | 0.52 | 68.2 | 87.0 | 0.26 | 89.3 | 65.6 | 0.07 |
|  | 1 | 16.0 | 23.6 |  | 27.3 | 13.0 |  | 7.1 | 31.3 |  |
|  | None or not relevant | 4.0 | 1.8 |  | 4.5 | 0.0 |  | 3.6 | 3.1 |  |
|  |  |  |  |  |  |  |  |  |  |  |
| Further diagnostic steps? Laboratory: Blood count / TSH / Creatinine / Liver tests | At least 3 | 26.0 | 63.6 | **<0.001** | 40.9 | 56.5 | 0.39 | 14.3 | 68.8 | **<0.001** |
|  | 1 or 2 | 64.0 | 36.4 |  | 54.6 | 43.5 |  | 71.4 | 31.2 |  |
|  | None or not relevant | 10.0 | 0.0 |  | 4.5 | 0.0 |  | 14.3 | 0.0 |  |
|  |  |  |  |  |  |  |  |  |  |  |
| Further diagnostic steps? Imaging: Chest X-ray / Abdominal scan / Chest CT scan | At least 2 | 58.0 | 65.4 | 0.71 | 54.6 | 65.2 | 0.19 | 60.7 | 65.6 | 0.50 |
|  | 1 | 34.0 | 29.1 |  | 31.8 | 34.8 |  | 35.7 | 25.0 |  |
|  | None or not relevant | 8.0 | 5.5 |  | 13.6 | 0.0 |  | 3.6 | 9.4 |  |
|  |  |  |  |  |  |  |  |  |  |  |
| After consulting laboratory and imaging results: Final diagnosis | Pulmonary neoplasia | 98.0 | 100.0 | 0.29 | 95.4 | 100.0 | 0.30 | 100.0 | 100.0 | 1.0 |
|  | Excludes endocrinology or hemato-oncology | 0.0 | 0.0 |  | 0.0 | 0.0 |  | 0.0 | 0.0 |  |
|  | Not relevant | 2.0 | 0.0 |  | 4.6 | 0.0 |  | 0.0 | 0.0 |  |
|  |  |  |  |  |  |  |  |  |  |  |
| General clinical reasoning | Good | 70.0 | 81.8 | 0.16 | 68.2 | 87.0 | 0.13 | 71.4 | 78.1 | 0.55 |
|  | Sufficient | 30.0 | 18.2 |  | 31.8 | 13.0 |  | 28.6 | 21.9 |  |
|  | Insufficient | 0.0 | 0.0 |  | 0.0 | 0.0 |  | 0.0 | 0.0 |  |

*P-values of chi-squared tests for each evaluation item. Bold indicates p <0.05.
Male SP: Male standardised patient, Female SP: Female standardised patient.
See **Appendix 2** for more details on content and evaluation criteria.

^a^

^a^

^b^

*

*

*

**

*

**

**Figure S4**. Evaluation of post-encounter communication.

^a^ Rated 1 (not at all) to 5 (totally)
^b^ Rated 1 (incompetent) to 5 (exceptionally competent)

* p < 0.05; ** p < 0.001

See **Appendix 2** for more details on content and evaluation criteria.
